# 2-Meth­oxy-4-methyl-1-[1-(phenyl­sulfon­yl)propan-2-yl]benzene

**DOI:** 10.1107/S1600536811035987

**Published:** 2011-09-14

**Authors:** Bao-Jun Shi, Li-Chun Ding, Gang Chen, Zhen-Ting Du

**Affiliations:** a State Key Laboratory of Crop Stress Biology for Arid Areas, Northwest A&F University, Yangling 712100, Shannxi Province, People’s Republic of China; b College of Science, Northwest A&F University, Yangling 712100, Shannxi Province, People’s Republic of China

## Abstract

The title mol­ecule, C_17_H_20_O_3_S, displays a U-shaped structure; the two benzene rings are nearly parallel and partially overlapped to each other, the dihedral angle and centroid-to-centroid distance being 15.0 (2)° and 3.723 (2) Å. In the crystal, weak inter­molecular C—H⋯O hydrogen bonds link the mol­ecules, forming supra­molecular chains running along the *a* axis.

## Related literature

For propargylic sulfides as precusors of indene derivatives, see: Peng *et al.* (2007 ▶). For a related structure, see: Xi *et al.* (2004 ▶).
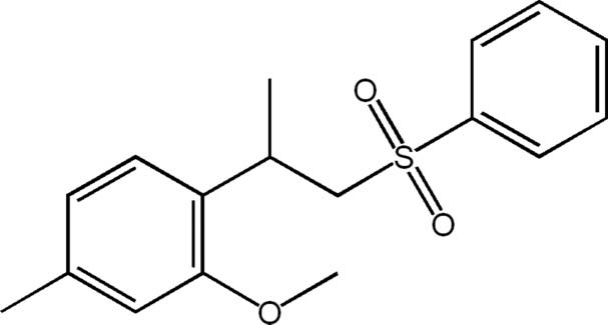


## Experimental

### 

#### Crystal data

C_17_H_20_O_3_S
*M* *_r_* = 304.39
Monoclinic, 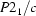

*a* = 9.101 (1) Å
*b* = 12.2579 (14) Å
*c* = 15.9251 (17) Å
β = 118.307 (1)°
*V* = 1564.1 (3) Å^3^
*Z* = 4
Mo *K*α radiation
μ = 0.21 mm^−1^
*T* = 298 K
0.42 × 0.27 × 0.13 mm

#### Data collection

Bruker SMART 1000 CCD area-detector diffractometer
Absorption correction: multi-scan (*SADABS*; Bruker, 2001 ▶) *T*_min_ = 0.915, *T*_max_ = 0.973
7654 measured reflections
2754 independent reflections
1641 reflections with *I* > 2σ(*I*)
*R*_int_ = 0.050

#### Refinement

*R*[*F* ^2^ > 2σ(*F* ^2^)] = 0.055
*wR*(*F* ^2^) = 0.187
*S* = 1.08
2754 reflections
193 parameters
H-atom parameters constrained
Δρ_max_ = 0.26 e Å^−3^
Δρ_min_ = −0.23 e Å^−3^

### 

Data collection: *SMART* (Bruker, 2007 ▶); cell refinement: *SAINT* (Bruker, 2007 ▶); data reduction: *SAINT*; program(s) used to solve structure: *SHELXTL* (Sheldrick, 2008 ▶); program(s) used to refine structure: *SHELXTL*; molecular graphics: *SHELXTL*; software used to prepare material for publication: *SHELXTL*.

## Supplementary Material

Crystal structure: contains datablock(s) global, I. DOI: 10.1107/S1600536811035987/xu5314sup1.cif

Structure factors: contains datablock(s) I. DOI: 10.1107/S1600536811035987/xu5314Isup2.hkl

Supplementary material file. DOI: 10.1107/S1600536811035987/xu5314Isup3.cml

Additional supplementary materials:  crystallographic information; 3D view; checkCIF report

## Figures and Tables

**Table 1:** Hydrogen-bond geometry (Å, °)

*D*—H⋯*A*	*D*—H	H⋯*A*	*D*⋯*A*	*D*—H⋯*A*
C11—H11*B*⋯O2^i^	0.96	2.59	3.503 (6)	159

Symmetry code: (i) 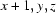.

**Fig. 1.**
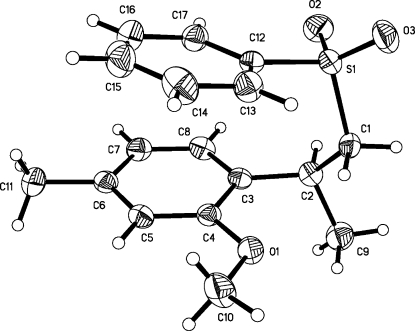
The molecular structure of the title compound with the atom numbering scheme. Displacement ellipsoids are drawn at the 30% probability level.

**Fig. 2.**
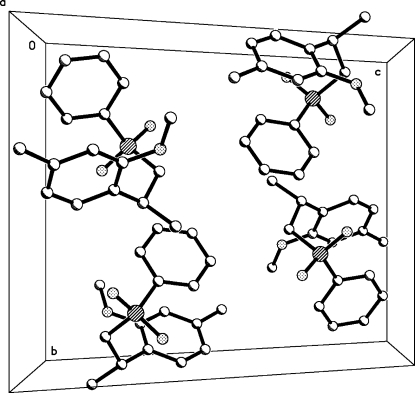
The molecular packing of (I) viewed along the a axis, with hydrogen bonds shown as dashed lines.

## supplementary crystallographic information

### Comment

Propargylic sulfides have been studied as the precusor of indene derivatives (Peng *et al.* 2007). As a result of our program of screening of new indene derivatives, we obtained a intermediate compound C_17_H_20_O_3_S (I) and the synthesis and structure are reported here.

There are two benzene rings in the title compound and they exhibit face-to-face conformation. The dihedral angle between the two benzene rings is 12.0 (2)°. The molecules of I are crystalized in *P*2_1_/*c* space group which is different from that of 2–phenyl–1–(*p*–toluenesulfonyl)propan–2–ol (*Pbca*, Xi *et al.*, 2004). In the crystal structure there is an intermolecular C—H···O hydrogen-bonding interaction (Table 1), which is helpful to the stabilization of the packing.

### Experimental

A mixture of 1-(1-bromopropan-2-yl)-4-methylbenzene (1.0 g, 4.7 mmol) and sodium benzenesulfinate (0.83 g, 5.6 mmol) in dry DMF (20 mL) was stirred over night at 80°C. When the reaction was completed, 50 mL water was added to the mixture and was extracted with ethyl acetate. The ethyl acetate layer was washed by 50 mL water, then 15 mL saturated sodium chloride and over anhydrous sodium sulfate and was separated on silica gel column chromatography with a gradient of petroleum ether and ethyl acetate as eluent to yield 1.3 g the title compound. The compound was then dissolved in ethyl acetate, and colorless crystals were formed on slow evaporation at room temperature over one week.

### Refinement

All H atoms were placed in geometrically calculated positions and refined using a riding model with C—H = 0.93 Å and N—H = 0.86 Å and with *U*_iso_(H) = 1.2*U*_eq_(C, N).

### Crystal data

**Table:**

C_17_H_20_O_3_S	*F*(000) = 648
*M**_r_* = 304.39	*D*_x_ = 1.293 Mg m^−^^3^
Monoclinic, *P*2_1_/*c*	Mo *K*α radiation, λ = 0.71073 Å
Hall symbol: -P 2ybc	Cell parameters from 1375 reflections
*a* = 9.101 (1) Å	θ = 2.5–23.0°
*b* = 12.2579 (14) Å	µ = 0.21 mm^−^^1^
*c* = 15.9251 (17) Å	*T* = 298 K
β = 118.307 (1)°	Block, colorless
*V* = 1564.1 (3) Å^3^	0.42 × 0.27 × 0.13 mm
*Z* = 4	

### Data collection

**Table:**

Bruker SMART 1000 CCD area-detector diffractometer	2754 independent reflections
Radiation source: fine-focus sealed tube	1641 reflections with *I* > 2σ(*I*)
graphite	*R*_int_ = 0.050
φ and ω scans	θ_max_ = 25.0°, θ_min_ = 2.5°
Absorption correction: multi-scan (*SADABS*; Bruker, 2001)	*h* = −10→10
*T*_min_ = 0.915, *T*_max_ = 0.973	*k* = −14→14
7654 measured reflections	*l* = −11→18

### Refinement

**Table:**

Refinement on *F*^2^	Primary atom site location: structure-invariant direct methods
Least-squares matrix: full	Secondary atom site location: difference Fourier map
*R*[*F*^2^ > 2σ(*F*^2^)] = 0.055	Hydrogen site location: inferred from neighbouring sites
*wR*(*F*^2^) = 0.187	H-atom parameters constrained
*S* = 1.08	*w* = 1/[σ^2^(*F*_o_^2^) + (0.0032*P*)^2^ + 0.4143*P*] where *P* = (*F*_o_^2^ + 2*F*_c_^2^)/3
2754 reflections	(Δ/σ)_max_ < 0.001
193 parameters	Δρ_max_ = 0.26 e Å^−^^3^
0 restraints	Δρ_min_ = −0.23 e Å^−^^3^

### Special details

**Table:**

Geometry. All esds (except the esd in the dihedral angle between two l.s. planes) are estimated using the full covariance matrix. The cell esds are taken into account individually in the estimation of esds in distances, angles and torsion angles; correlations between esds in cell parameters are only used when they are defined by crystal symmetry. An approximate (isotropic) treatment of cell esds is used for estimating esds involving l.s. planes.
Refinement. Refinement of F^2^ against ALL reflections. The weighted R-factor wR and goodness of fit S are based on F^2^, conventional R-factors R are based on F, with F set to zero for negative F^2^. The threshold expression of F^2^ > 2sigma(F^2^) is used only for calculating R-factors(gt) etc. and is not relevant to the choice of reflections for refinement. R-factors based on F^2^ are statistically about twice as large as those based on F, and R- factors based on ALL data will be even larger.

### Fractional atomic coordinates and isotropic or equivalent isotropic displacement parameters (Å^2^)

**Table:**

	*x*	*y*	*z*	*U*_iso_*/*U*_eq_	
O1	0.9829 (3)	0.3534 (2)	0.33678 (17)	0.0512 (7)	
O2	0.3680 (3)	0.4052 (2)	0.16790 (19)	0.0622 (8)	
O3	0.4268 (4)	0.2682 (2)	0.2938 (2)	0.0677 (9)	
S1	0.48398 (11)	0.33304 (8)	0.23977 (7)	0.0450 (3)	
C1	0.6540 (4)	0.4104 (3)	0.3246 (2)	0.0419 (9)	
H1A	0.6215	0.4415	0.3693	0.050*	
H1B	0.7460	0.3608	0.3601	0.050*	
C2	0.7182 (4)	0.5027 (3)	0.2863 (3)	0.0431 (9)	
H2	0.6230	0.5508	0.2496	0.052*	
C3	0.7823 (4)	0.4663 (3)	0.2192 (2)	0.0372 (9)	
C4	0.9128 (4)	0.3909 (3)	0.2449 (3)	0.0394 (9)	
C5	0.9644 (4)	0.3587 (3)	0.1804 (3)	0.0434 (9)	
H5	1.0517	0.3091	0.1993	0.052*	
C6	0.8891 (4)	0.3985 (3)	0.0877 (3)	0.0429 (9)	
C7	0.7631 (4)	0.4745 (3)	0.0628 (3)	0.0470 (10)	
H7	0.7123	0.5040	0.0016	0.056*	
C8	0.7120 (4)	0.5070 (3)	0.1277 (3)	0.0453 (10)	
H8	0.6270	0.5583	0.1091	0.054*	
C9	0.8460 (5)	0.5702 (3)	0.3700 (3)	0.0619 (12)	
H9A	0.9400	0.5253	0.4095	0.093*	
H9B	0.7955	0.5973	0.4069	0.093*	
H9C	0.8825	0.6305	0.3460	0.093*	
C10	1.1077 (5)	0.2713 (4)	0.3645 (3)	0.0709 (13)	
H10A	1.0622	0.2083	0.3246	0.106*	
H10B	1.1445	0.2514	0.4298	0.106*	
H10C	1.2005	0.2987	0.3579	0.106*	
C11	0.9425 (5)	0.3583 (4)	0.0172 (3)	0.0630 (12)	
H11A	0.9019	0.2854	−0.0021	0.095*	
H11B	1.0621	0.3586	0.0463	0.095*	
H11C	0.8975	0.4053	−0.0376	0.095*	
C12	0.5631 (4)	0.2425 (3)	0.1847 (3)	0.0381 (9)	
C13	0.6598 (5)	0.1548 (3)	0.2355 (3)	0.0529 (10)	
H13	0.6851	0.1441	0.2987	0.063*	
C14	0.7178 (6)	0.0837 (3)	0.1916 (4)	0.0674 (13)	
H14	0.7839	0.0248	0.2255	0.081*	
C15	0.6790 (6)	0.0988 (4)	0.0979 (4)	0.0694 (13)	
H15	0.7183	0.0496	0.0687	0.083*	
C16	0.5828 (5)	0.1856 (4)	0.0468 (3)	0.0628 (12)	
H16	0.5575	0.1958	−0.0165	0.075*	
C17	0.5239 (5)	0.2579 (3)	0.0910 (3)	0.0481 (10)	
H17	0.4578	0.3168	0.0572	0.058*	

### Atomic displacement parameters (Å^2^)

**Table:**

	*U*^11^	*U*^22^	*U*^33^	*U*^12^	*U*^13^	*U*^23^
O1	0.0449 (15)	0.0676 (16)	0.0393 (15)	0.0191 (13)	0.0185 (13)	0.0142 (13)
O2	0.0437 (16)	0.0711 (17)	0.0598 (18)	0.0197 (13)	0.0147 (15)	0.0003 (15)
O3	0.074 (2)	0.0761 (18)	0.077 (2)	−0.0205 (16)	0.0555 (18)	−0.0102 (17)
S1	0.0406 (6)	0.0526 (6)	0.0480 (6)	−0.0027 (4)	0.0259 (5)	−0.0040 (5)
C1	0.044 (2)	0.0461 (19)	0.039 (2)	0.0018 (16)	0.0228 (18)	−0.0041 (17)
C2	0.045 (2)	0.0388 (19)	0.047 (2)	0.0029 (16)	0.0223 (19)	0.0027 (17)
C3	0.0347 (19)	0.0327 (17)	0.043 (2)	−0.0027 (15)	0.0171 (18)	0.0024 (16)
C4	0.036 (2)	0.043 (2)	0.037 (2)	−0.0035 (16)	0.0158 (18)	0.0027 (17)
C5	0.036 (2)	0.045 (2)	0.048 (2)	0.0028 (16)	0.0190 (19)	0.0011 (18)
C6	0.040 (2)	0.046 (2)	0.044 (2)	−0.0085 (16)	0.0200 (18)	−0.0016 (17)
C7	0.046 (2)	0.054 (2)	0.036 (2)	−0.0035 (18)	0.0164 (19)	0.0102 (18)
C8	0.042 (2)	0.041 (2)	0.051 (2)	0.0014 (16)	0.021 (2)	0.0116 (18)
C9	0.069 (3)	0.054 (2)	0.065 (3)	−0.009 (2)	0.034 (3)	−0.012 (2)
C10	0.058 (3)	0.095 (3)	0.063 (3)	0.039 (2)	0.032 (2)	0.035 (3)
C11	0.067 (3)	0.082 (3)	0.046 (2)	−0.005 (2)	0.031 (2)	−0.008 (2)
C12	0.035 (2)	0.0393 (18)	0.040 (2)	−0.0058 (15)	0.0178 (17)	−0.0037 (17)
C13	0.054 (2)	0.057 (2)	0.049 (2)	0.0062 (19)	0.025 (2)	0.008 (2)
C14	0.067 (3)	0.047 (2)	0.097 (4)	0.013 (2)	0.046 (3)	0.007 (3)
C15	0.079 (3)	0.058 (3)	0.085 (4)	0.000 (2)	0.050 (3)	−0.017 (3)
C16	0.070 (3)	0.072 (3)	0.052 (3)	−0.004 (2)	0.033 (2)	−0.012 (2)
C17	0.049 (2)	0.051 (2)	0.043 (2)	−0.0013 (18)	0.021 (2)	0.0019 (19)

### Geometric parameters (Å, °)

**Table:**

O1—C4	1.369 (4)	C8—H8	0.9300
O1—C10	1.422 (4)	C9—H9A	0.9600
O2—S1	1.435 (3)	C9—H9B	0.9600
O3—S1	1.438 (3)	C9—H9C	0.9600
S1—C12	1.766 (4)	C10—H10A	0.9600
S1—C1	1.771 (3)	C10—H10B	0.9600
C1—C2	1.527 (5)	C10—H10C	0.9600
C1—H1A	0.9700	C11—H11A	0.9600
C1—H1B	0.9700	C11—H11B	0.9600
C2—C3	1.507 (5)	C11—H11C	0.9600
C2—C9	1.530 (5)	C12—C17	1.372 (5)
C2—H2	0.9800	C12—C13	1.382 (5)
C3—C8	1.378 (5)	C13—C14	1.370 (6)
C3—C4	1.404 (5)	C13—H13	0.9300
C4—C5	1.375 (5)	C14—C15	1.374 (6)
C5—C6	1.389 (5)	C14—H14	0.9300
C5—H5	0.9300	C15—C16	1.373 (6)
C6—C7	1.382 (5)	C15—H15	0.9300
C6—C11	1.501 (5)	C16—C17	1.388 (5)
C7—C8	1.379 (5)	C16—H16	0.9300
C7—H7	0.9300	C17—H17	0.9300
			
C4—O1—C10	117.9 (3)	C2—C9—H9A	109.5
O2—S1—O3	118.67 (18)	C2—C9—H9B	109.5
O2—S1—C12	108.05 (17)	H9A—C9—H9B	109.5
O3—S1—C12	107.38 (16)	C2—C9—H9C	109.5
O2—S1—C1	108.94 (16)	H9A—C9—H9C	109.5
O3—S1—C1	105.84 (17)	H9B—C9—H9C	109.5
C12—S1—C1	107.48 (17)	O1—C10—H10A	109.5
C2—C1—S1	117.0 (2)	O1—C10—H10B	109.5
C2—C1—H1A	108.0	H10A—C10—H10B	109.5
S1—C1—H1A	108.0	O1—C10—H10C	109.5
C2—C1—H1B	108.0	H10A—C10—H10C	109.5
S1—C1—H1B	108.0	H10B—C10—H10C	109.5
H1A—C1—H1B	107.3	C6—C11—H11A	109.5
C3—C2—C1	114.4 (3)	C6—C11—H11B	109.5
C3—C2—C9	112.8 (3)	H11A—C11—H11B	109.5
C1—C2—C9	109.2 (3)	C6—C11—H11C	109.5
C3—C2—H2	106.6	H11A—C11—H11C	109.5
C1—C2—H2	106.6	H11B—C11—H11C	109.5
C9—C2—H2	106.6	C17—C12—C13	120.6 (4)
C8—C3—C4	116.8 (4)	C17—C12—S1	119.8 (3)
C8—C3—C2	120.0 (3)	C13—C12—S1	119.6 (3)
C4—C3—C2	123.2 (3)	C14—C13—C12	119.3 (4)
O1—C4—C5	123.4 (3)	C14—C13—H13	120.4
O1—C4—C3	115.7 (3)	C12—C13—H13	120.4
C5—C4—C3	120.9 (3)	C13—C14—C15	120.4 (4)
C4—C5—C6	121.5 (3)	C13—C14—H14	119.8
C4—C5—H5	119.3	C15—C14—H14	119.8
C6—C5—H5	119.3	C16—C15—C14	120.7 (4)
C7—C6—C5	117.8 (4)	C16—C15—H15	119.6
C7—C6—C11	121.7 (4)	C14—C15—H15	119.6
C5—C6—C11	120.5 (3)	C15—C16—C17	119.1 (4)
C8—C7—C6	120.6 (4)	C15—C16—H16	120.5
C8—C7—H7	119.7	C17—C16—H16	120.5
C6—C7—H7	119.7	C12—C17—C16	119.9 (4)
C3—C8—C7	122.4 (3)	C12—C17—H17	120.0
C3—C8—H8	118.8	C16—C17—H17	120.0
C7—C8—H8	118.8		
			
O2—S1—C1—C2	−39.9 (3)	C5—C6—C7—C8	−1.6 (5)
O3—S1—C1—C2	−168.6 (3)	C11—C6—C7—C8	177.5 (3)
C12—S1—C1—C2	76.9 (3)	C4—C3—C8—C7	1.4 (5)
S1—C1—C2—C3	−60.9 (4)	C2—C3—C8—C7	−178.4 (3)
S1—C1—C2—C9	171.6 (3)	C6—C7—C8—C3	−0.1 (5)
C1—C2—C3—C8	121.4 (3)	O2—S1—C12—C17	7.0 (3)
C9—C2—C3—C8	−112.9 (4)	O3—S1—C12—C17	136.1 (3)
C1—C2—C3—C4	−58.5 (4)	C1—S1—C12—C17	−110.4 (3)
C9—C2—C3—C4	67.2 (4)	O2—S1—C12—C13	−171.0 (3)
C10—O1—C4—C5	−4.4 (5)	O3—S1—C12—C13	−41.9 (3)
C10—O1—C4—C3	175.7 (3)	C1—S1—C12—C13	71.6 (3)
C8—C3—C4—O1	178.7 (3)	C17—C12—C13—C14	0.7 (6)
C2—C3—C4—O1	−1.4 (5)	S1—C12—C13—C14	178.7 (3)
C8—C3—C4—C5	−1.2 (5)	C12—C13—C14—C15	−0.7 (6)
C2—C3—C4—C5	178.7 (3)	C13—C14—C15—C16	0.5 (7)
O1—C4—C5—C6	179.6 (3)	C14—C15—C16—C17	−0.5 (7)
C3—C4—C5—C6	−0.5 (5)	C13—C12—C17—C16	−0.7 (5)
C4—C5—C6—C7	1.9 (5)	S1—C12—C17—C16	−178.6 (3)
C4—C5—C6—C11	−177.3 (3)	C15—C16—C17—C12	0.5 (6)

### Hydrogen-bond geometry (Å, °)

**Table:**

*D*—H···*A*	*D*—H	H···*A*	*D*···*A*	*D*—H···*A*
C11—H11B···O2^i^	0.96	2.59	3.503 (6)	159

Symmetry codes: (i) *x*+1, *y*, *z*.
